# The impact of previous health on the mortality after aneurysmal subarachnoid hemorrhage: analysis of a prospective Swedish multicenter study

**DOI:** 10.1007/s00701-022-05464-8

**Published:** 2023-01-12

**Authors:** Elisabeth Ronne Engström, Bryndís Baldvinsdóttir, Helena Aineskog, Peter Alpkvist, Per Enblad, Johanna Eneling, Steen Fridriksson, Jan Hillman, Paula Klurfan, Erik Kronvall, Peter Lindvall, Ann-Christin Von Vogelsang, Ola G. Nilsson, Mikael Svensson

**Affiliations:** 1 Neurosurgery, Medical Sciences, Uppsala University, Uppsala, Sweden; 2 Neurosurgery, Clinical Sciences, Lund University, Lund, Sweden; 3 Neurosurgery, Clinical Sciences, Umea University, Umeå, Sweden; 4 Neurosurgery, Clinical Neuroscience, Karolinska Institute, Stockholm, Sweden; 5 Neurosurgery, Biomedical and Clinical Sciences, Linköping University, Linköping, Sweden; 6 Neurosurgery, Clinical Neuroscience, Gothenburg University, Gothenburg, Sweden

**Keywords:** Spontaneous subarachnoid hemorrhage, Mortality, Previous health, Medical conditions, National prospective study

## Abstract

**Purpose:**

There is an an increasing awareness of the importance of health and lifestyle for stroke diseases like spontaneous subarachnoid hemorrhage (SAH). However, the importance of pre-existing medical conditions for clinical course and mortality after SAH has not been studied. The aim of the present study was to identify pre-existing conditions contributing to mortality after SAH.

**Methods:**

Data were extracted from a Swedish national prospective study on patients with SAH. Variables were defined for age, sex, body mass index (BMI), clinical condition at admission, and for 10 pre-existing medical conditions. Models predicting mortality in three time intervals with all possible subsets of these variables were generated, compared and selected using Akaike’s information criterion.

**Results:**

1155 patients with ruptured aneurysms were included. The mortality within 1 week was 7.6%, 1 month 14.3%, and 1 year 18.7%. The most common pre-existing medical conditions were smoking (57.6%) and hypertension (38.7%). The model’s best predicting mortality within 1 week and from 1 week to 1 month included only the level of consciousness at admission and age, and these two variables were present in all the models among the top 200 in Akaike score for each time period. The most predictive model for mortality between 1 month and 1 year added previous stroke, diabetes, psychiatric disease, and BMI as predictors.

**Conclusion:**

Mortality within the first month was best predicted simply by initial level of consciousness and age, while mortality within from 1 month to 1 year was significantly influenced by pre-existing medical conditions.

## Introduction

Spontaneous subarachnoid hemorrhage (SAH) constitutes 5% of stroke incidents. Compared to other stroke patients, the mean age in SAH is lower and the short-term mortality is higher. In three out of four cases, SAH is caused by the rupture of an intracranial aneurysm and repeated bleedings are then associated with a high mortality. Therefore, once a SAH has occurred, most patients are referred to a neurosurgical center to diagnose and treat a ruptured aneurysm with surgery or neurointervention, and to prevent and treat complications to the hemorrhage.

Patients with SAH often perceive themselves as having been previously healthy until they suddenly suffer from an aneurysm rupture. However, there is an increasing awareness of the importance of pre-existing medical conditions for development of SAH. Similar conditions have been suggested for the development and rupture of a brain aneurysm as for acquiring ischemic as well as other types of hemorrhagic stroke. It has been suggested that more than 90% of the incidents of stroke are attributable to modifiable conditions including lifestyle and metabolic factors [4]. For SAH, studies suggest that hypertension, smoking, and alcohol use could be involved (for references, see [8].

The bulk of the mortality after SAH occurs in the acute phase, presumably because of the brain injury from the first bleeding, but also caused by other acute complications. There are studies on the cause of death after the acute phase, and in the short term, this correlated to cardiovascular incidents [11]. In the long term, mortality correlated with cerebrovascular disorders [10]. However, there are few or no studies on the impact of the earlier health and lifestyle on the mortality after SAH.

Earlier health, pre-existing medical conditions, as well as lifestyle factors, have in common that they are difficult to quantify, especially in reviewing medical records for retrospective studies. The course of an acute disease like SAH can be dramatic, and the focus of the medical records is on the acute situation rather than the patients’ previous health problems. The absence of information on a previous condition does not mean it did not exist.

The aim of the present study was therefore to use systematically collected data from a prospective national Swedish study on SAH to describe the pattern of pre-existing medical conditions and determine their impact on mortality during three different time intervals: the first week following hemorrhage, 1 week to 1 month, and 1 month to 1 year.

## Material and methods

The data used in this study were taken from a prospective study involving Sweden’s six neurosurgical departments, which are in Gothenburg, Linköping, Lund, Stockholm, Umeå, and Uppsala. The inclusion criterion was admission to a neurosurgical ward due to a spontaneous subarachnoid hemorrhage between Sept 1st 2014 and March 31st 2018. Patients that were not admitted to neurosurgical wards were not included. The planning of the study, defining variables, and setting up the study protocol were done by a steering group consisting of vascular neurosurgeons from all the departments.

Data were entered in an electronic case report form (CRF) with coded patient identifiers. The CRF consisted of eight modules: 1. basic demographic information, 2. previous medical history and events before admission to neurosurgery, 3. neurological assessments, 4. radiological information, 5. information about the aneurysms, 6. neurosurgical and endovascular treatment, 7. neuro-intensive care, 8. 1-year follow-up of the aneurysm and the functional outcome and health-related quality of life. In the present paper, data from modules 1, 2, and 3 were used.

The demographic data and previous medical history were reported by the patient, the next of kin or extracted from existing medical records. The pre-existing medical conditions recorded were hypertension, cardiac disease, diabetes type 1 or 2, earlier stroke, psychiatric disease, and any type of kidney disease. A condition was considered present if it had been diagnosed before the SAH and the patient was undergoing treatment or follow-up for it. We also noted age, sex, height, weight, smoking and snuff tobacco history, use of alcohol, and heredity for SAH, cerebral aneurysms, or related connective tissue diseases. The medical conditions were noted as “yes/no/unknown” except for smoking that was graded as “never smoked,” “stopped smoking > 6 months before the SAH,” or “ongoing smoking or stopped < 6 months before SAH”. Body mass index (BMI) was calculated from the patients’ height and weight. For the neurological and clinical assessment, the grading scales Reaction Level Score (RLS-85) [16], Glasgow Coma Score (GCS) [17], Hunt and Hess [7], and World Federation of Neurosurgical Societies (WFNS) [18] were used. Focal neurological deficits including pupillary light reactions were also noted. After 1 year, a follow-up was done and, in case of death, the day of death was noted.

### Statistics

A total of 12 variables, all with less than 10% missing or unknown values, were included in the analyses. The following were included as dichotomized predictive variables: cardiac disease, hypertension, diabetes (type 1 or 2), earlier stroke, kidney disease, psychiatric disease, smoking, alcohol use, and sex. BMI and age were included as continuous variables. In addition, the first documented neurological condition was used. Of the methods of assessing neurological condition, RLS-85 had the least amount of missing data and was therefore used in the calculations. RLS-85 was dichotomized as conscious (RLS 1–3) and unconscious (RLS 4–8).

Statistica 13 was used for descriptive and comparative statistics. The influence of pre-existing medical conditions on the mortality during the first week after the rupture, between 1 week and 1 month, and between 1 month and 1 year, was evaluated with general linear/nonlinear modelling of best subsets of variables according to Akaike’s information criterion (AIC) [20]. For the three time periods considered, Statistica evaluated models for every possible subset of variables and returned the top 200 of the 4096 possible models as ranked by AIC. We have presented the top three models for each time period. The relative likelihood of a model compared to the best (minimum AIC) model was estimated using the formula exp((AIC_min_ – AIC_model_)/2) [19]. For example, a relative likelihood of 0.5 means that the model is half as likely to be the best model as is the top ranked model. A *p* value of < 0.05 was considered significant.

### Ethical permission

The Regional Ethical Review Board granted permission for this study.

## Results

Data from the 1155 SAH patients with a diagnosed aneurysm (saccular, dissection, fusiform, or flow related in association with an AVM) were included in this study. 67.8% were women. Mean age was 58.1 ± 13.3 years and BMI median 25.7. On admission, 97.7% of patients had their neurological condition assessed, the majority during the admission to the neurosurgical department and the rest at the first hospital. Median WFNS was 2 and 8.6% had one or two fixed pupils. For the first neurological assessment, missing values for RLS-85, Hunt and Hess, GCS, WFNS, and pupils ranged from 3.3% (pupils) to 5% (GCS) (Table 1).

**Table 1:** Basic demographic information (*n* = 1155)

Age (mean, SD)	58.1 ± 13.3	
Women	67.8%	
	Median (IQR)	Missing values (%)
BMI	25.7 (22.9; 29.3)	9.0
First RLS-85	2 (1;3)	3.9
First GCS	11 (8;15)	5.0
First Hunt and Hess	2 (2; 4)	4.6
First WFNS	2 (1;4)	5.0
One or two fixed pupils	8.6%	3.3

The aneurysm treatments were 63.3% endovascular, 27.1% surgical, and 1.8% with both techniques. In 7.8% of patients, no treatment was done due to poor clinical condition, complex aneurysm morphology in combination with advanced age, or due to spontaneous occlusion. The mortality within 1 week from rupture was 7.6%, within 1 month 14.3%, and 1 year 18.7%.

**Fig. 1:**
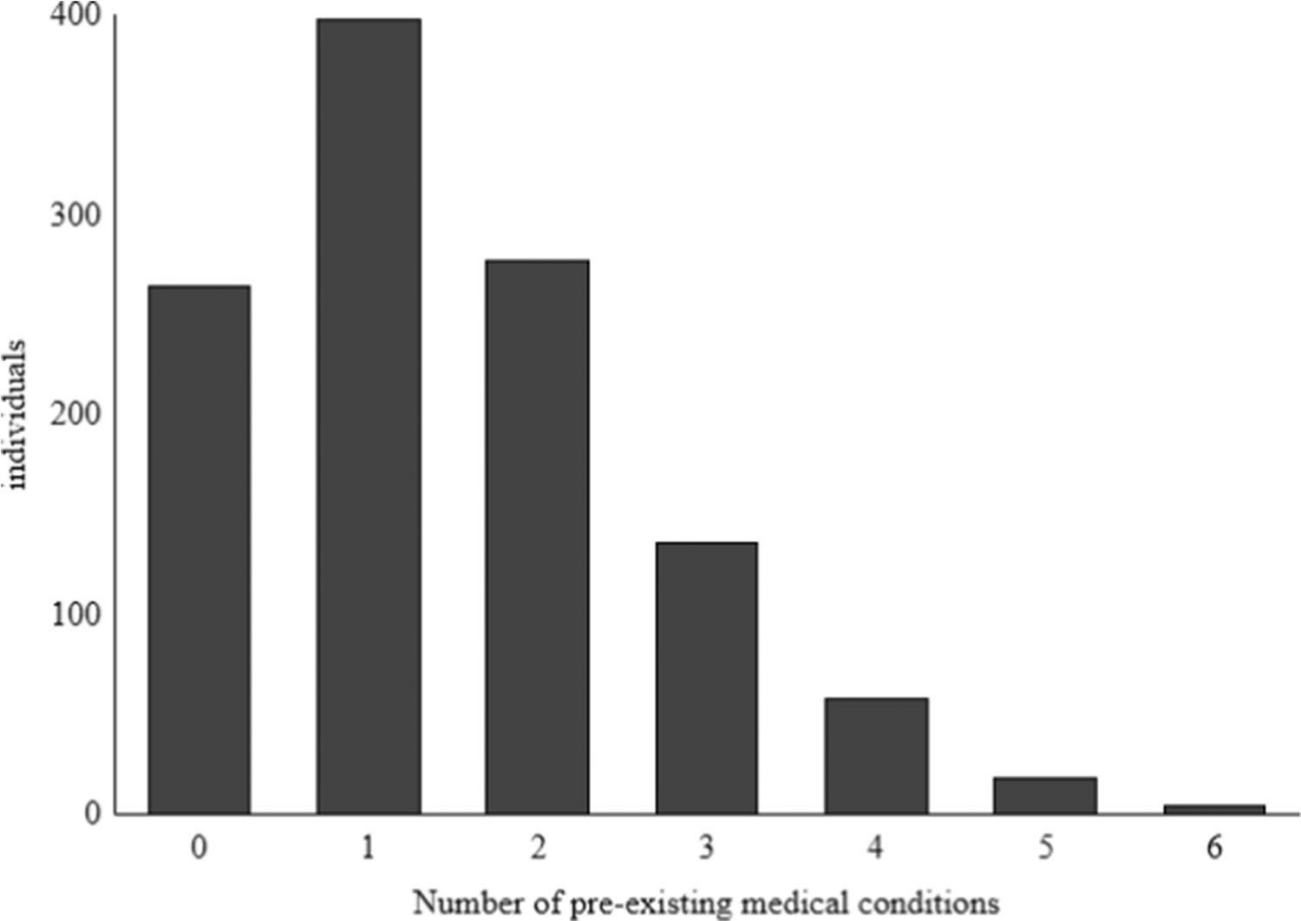
This figure shows the distribution of the numbers of pre-existing medical conditions

The most common pre-existing medical condition was smoking, either ongoing or earlier, which applied to 57.6% of included patients. This was followed by hypertension which was seen in 38.7% (Table 2). Two or more medical conditions were recorded at admission in 43.0% of the cohort (Fig. 1).

**Table 2:** Pre-existing medical condition

	Yes (%)	Missing or unknown (%)
Smoking, ongoing, or earlier	57.6	10.0
Hypertension	38.7	1.2
Alcohol use	18.2	14.0
Cardiac disease	9.4	0.8
Psychiatric disease	8.6	1.6
Heredity for SAH/aneurysm or related disease	7.8	18.6
Snuff tobacco	7.0	14.5
Earlier stroke	4.7	1.4
Diabetes 1 and 2	4.5	1.0
Kidney disease	2.4	1.1

The models selected based on the Akaike criterion for the three time periods are presented in Table 3, with the selected variables, the statistical significance of the model, and the relative likelihood compared to the best model for the same time period. The model’s best predicting mortality within 1 week and from 1 week to 1 month included only the level of consciousness at admission and age, and these two variables were present in all the models among the top 200 in Akaike score for each time period. The most predictive model for mortality between 1 month and 1 year added previous stroke, diabetes, psychiatric disease, and BMI as predictors.

**Table 3:** Models predicting mortality: predictor variables, model likelihood relative to the best (minimum AIC) model, and statistical significance. RLS-85 is the reaction level score used to evaluate level of consciousness on admission

Mortality within 1 week	Relative likelihood	*p* value
RLS-85, age	1	1.22 10 − ^15^
RLS-85, cardiac dis., age	0.778	3.77 10 − ^15^
RLS-85, psychiatric dis., age	0.734	4.00 10 − ^15^
Mortality between 1 week and 1 month		
RLS-85, age	1	1.01 10 − ^06^
RLS-85, age, cardiac dis	0.764	2.17 10 − ^06^
RLS-85, age, psychiatric dis	0.640	2.58 10 − ^06^
Mortality between 1 month and 1 year		
RLS-85, diabetes, earlier stroke, psychiatric disease, age	1	3.53 10 − ^05^
RLS-85, diabetes, cardiac dis., earlier stroke, psychiatric dis., age	0.959	3.96 10 − ^05^
RLS-85, diabetes, cardiac dis., earlier stroke, psychiatric dis., age, BMI	0.747	5.06 10 − ^05^

## Discussion

The study presents for the first time the results from this prospective Swedish national study on patients hospitalized due to spontaneous SAH. In the present paper, we focused on pre-existing medical conditions. We related these to mortality during three different time periods, since we hypothesized that the role of pre-existing conditions would change during the course of the disease. Within 1 week from rupture, the mortality was 7.6%. This is on the expected level based on national experience [1, 14, 15]. After 1 month, the mortality was 14.3%. This can be compared to a large population-based study with 19% 28-day mortality [2].

Models with variables for predicting mortality for the time periods, the first week, 1 week to 1 month, and 1 month to 1 year following rupture, were tested. The three top models selected for the first two time periods were identical, and the relative likelihoods assigned very similar (Table 3). The top rated model in both time periods, by a wide margin, consisted simply of level of consciousness and age. This is an expected finding since the impact of the initial bleeding can be severe and those in too bad neurological condition at admission are often not treated for ruptured aneurysms. Even if treated, however, for patients in very poor neurological condition on admission, the initial bleeding is likely to lead to serious cerebral complications that can be fatal without regard to previous medical conditions.

The second and third best predicting models for mortality in the first two time periods also included earlier cardiac and psychiatric disease. Cardiovascular events have been found to correlate with increased short-term mortality after SAH [11]. Possibly cardiac disease is an expression of a more generalized artherosclerotic disease. This could be supported by a study [2] in which the authors found an increased risk both for recurrent stroke and major vascular events during a 5-year period after SAH. The mechanism of influence of earlier psychiatric disease is not clear. An association between depression and cardiac disease is known [6]. Several mechanisms for this have been suggested, e.g., inflammation, disturbed platelet function, and the influence of drug therapy. Behavioral factors possibly preventing individuals from necessary somatic care should also be considered [6]. Vascular health in patients with psychiatric conditions should be further studied.

In the best predictive models of the mortality for those that survived the first month but died within 1 year, diabetes type 1 or 2, previous stroke, and BMI were added (Table 3). It seems that after surviving the impact of the bleeding and cerebral complications in the acute phase, conditions of other organs become increasingly important for survival. Diabetes could be a contributing factor to the long-term vascular mortality in some cases. Earlier publications have shown that SAH patients have an increased mortality due to cerebrovascular disorders [10, 9]. The influence of previous stroke on mortality has not been addressed before, but this finding suggests that SAH may be part of a more systemic vascular disorder.

In our patient material, 43% had two or more pre-existing medical conditions. We found that smoking, at any time, and hypertension were the most common. These two factors, however, were not included in the best models predicting mortality. This could be an effect of a public awareness in Sweden that hypertension should be treated. It is also possible that, by the time of the rupture of the aneurysm, the vascular injury caused by hypertension and smoking had already manifested in cardiac disease, which was included in the best predicting models for mortality, at all three time intervals. In our study, we dichotomized the information on smoking in the statistical analysis to “never smoked” or “smoked at some time” which also could lead to loosing statistical power on the effect of smoking. However, smoking is difficult to quantify, especially considering how much environmental smoking “non-smokers” have sometimes been exposed to. Another possibility for smoking not ending up in the best predicting models could be that the rate of smoking has decreased in Sweden. Between 2004 and 2007, 15% reported daily smoking and in 2018–2021, it was 7% [21].

BMI was only included in the third best model for mortality between 1 month and 1 year. The effect of BMI on the course of the disease has been debated. In one retrospective study [5], the effect of obesity defined as BMI ≥ 30, diabetes, and hypertension on 30-day outcome was assessed in a cohort of surgically treated patients with SAH. They found that diabetes increased the risk for respiratory adverse events and death, hypertension increased the risk for cerebrovascular incidents, but that obesity was not associated with adverse outcomes. In a multicenter study, increased BMI appeared to have a protective effect on rupture in men over 50 years old [3]. However, in a review, it has also been concluded that the studies published so far could not provide convincing proof about this [12]. In fact, there was evidence that high BMI increased the risk for poor outcome in good grade aneurysmal SAH [13]. High BMI is generally thought to increase the risk for ischemic and hemorrhagic stroke [4]. However, there is no clear information in the literature about what levels of BMI that are associated with increased risk. We suggest instead that the medical effects of obesity, e.g., hypertension and diabetes, are more important for cerebrovascular diseases than the exact BMI per se, but this must be further studied.

### Methodological strengths and limitations

A strength of our study is that it is prospective and nationwide and that we have structured and verified information regarding multiple pre-existing conditions for all cases in this large, multicenter dataset. Our results are in line with earlier studies. This could be viewed as a limitation in that the results lack “novelty.” On the other hand, the study was properly conducted and reported. Demonstrating the reproducibility of scientific results in a range of studies is important in all fields.

One weakness of the present study is the definition of the pre-existing medical conditions. However, our intention was that these definitions should act as a screening for ongoing health problems. Obviously, some disease groups, e.g., cardiac disease or psychiatric disease, could have been further subdivided and detailed. Another possible weakness is that the study is done on patients referred to neurosurgical units. Patients who for various reasons stay at the first hospitals were not included. In Sweden, health care is tax-financed and organized in geographical regions with the neurosurgical units located in the larger central hospitals. The normal procedure for a SAH is that the patient is referred to a neurosurgical center unless there are clear medical reasons to not do this. Since 2020, SAH has been included in the Swedish national quality register for Stroke (Riksstroke), and patients with spontaneous SAH who are not referred to a neurosurgical center are also registered. The rate of registration is continuously improving, but from the year report 2021 [22], it can be estimated that 77% were sent to a neurosurgical unit. Those that stayed in the first hospital were significantly older, median 79 years vs 59.5 years for those admitted. The reasons for not sending the patients to neurosurgery were bad neurological condition, other medical issues, and age, alone or in combination. Sometimes, radiology at home hospital was enough and no more investigation was felt necessary. It seems that the patients with SAH who are not referred to neurosurgical units are as a group very different from those who are referred. Our results and conclusions concern the group with aneurysmal SAH admitted to neurosurgery.

Different statistical methods could be used. We prefer to present the results from the multivariate analysis through model building. The main reason is that a set of variables resembles the clinical reality. Efforts to break out single variables and compare them to each other carries the risk of overestimating their clinical significance in isolation from the overall clinical picture.

## Conclusion

This study presents the initial results from a national prospective study on patients with SAH and that were referred to neurosurgery. The mortality within the first week was 7.6%, at 1 month 14.3%, and 1 year 18.7%. Based on the Akaike information criterion, the models that best predicted mortality during the first week and from 1 week to 1 month consisted simply of level of consciousness on admission and age. Between 1 month and 1 year, diabetes, earlier stroke, psychiatric disease, cardiac disease, and BMI were also important predictors of mortality and were included in the highest ranked models. Therefore, our data indicate that mortality within 1 month is dominated by the severity of the SAH and age, while these pre-existing medical conditions become important risk factors for mortality in the period from 1 month to 1 year following rupture.
